# FACTORS ASSOCIATED WITH ASTHMA IN BRAZILIAN ADOLESCENTS: NATIONAL ADOLESCENT SCHOOL-BASED HEALTH SURVEY (PENSE-2012)

**DOI:** 10.1590/1984-0462/;2019;37;4;00002

**Published:** 2019-07-18

**Authors:** Bianca Caroline Elias, Janiquelli Barbosa Silva, Laís Amaral Mais, Sarah Warkentin, Tulio Konstantyner, Dirceu Solé

**Affiliations:** a Universidade Federal de São Paulo, São Paulo, SP, Brazil.

**Keywords:** Asthma, Adolescent, Epidemiological surveys, Logistic models, Adolescente, Asma, Inquéritos epidemiológicos, Modelos logísticos

## Abstract

**Objective:**

To identify factors associated with asthma in Brazilian adolescents.

**Methods:**

Cross-sectional study based on data from the 2012 National Adolescent School-based Health Survey (PeNSE), a Brazilian survey applied by a self-reported questionnaire in a representative sample of 9^th^-grade students. Descriptive and inferential analysis was made based on the demographic, socioeconomic, clinical, food consumption and environmental characteristics potentially associated with asthma. Adolescents who presented wheezing in the last 12 months were considered asthmatic. A multiple logistic regression model was adjusted for confounding factors. Significance was defined as p≤0.05.

**Results:**

A total of 106,983 adolescents were studied. The prevalence of asthma was 23.2%. The final model was composed of 11 variables that were independently associated with asthma: female sex (OR=1.17), <14 years old (OR=1.12), not living with parents (OR=1.06), the highest number of days consuming ultra-processed foods (OR=1.16), lunch or dinner time without presence of parents or guardians (OR=1.13), meals in front of the TV or while studying (OR=1.18), not having breakfast frequently (OR=1.22), having smoked cigarettes (OR=1.36), having tried alcoholic beverage (OR=1.37), having used illicit drugs (OR=1.29) and having sought health care in the last year (OR=1.67).

**Conclusions:**

The results of the present study reinforce the multifactorial characteristic of asthma diagnosis. Prevention and control strategies should focus on groups of adolescents living in inadequate conditions when it comes to family dynamics, food consumption and behavior (drug use).

## RESUMO

**Objetivo:**

Identificar fatores associados à asma em adolescentes brasileiros.

**Métodos:**

Estudo transversal baseado em dados da Pesquisa Nacional de Saúde do Escolar (PeNSE-2012), que foi um inquérito brasileiro realizado por meio de questionário autoaplicável em amostra representativa de alunos do 9º ano do ensino fundamental. Foi realizada análise descritiva e inferencial das características demográficas, socioeconômicas, clínicas, alimentares e ambientais potencialmente ligadas à asma. Foram considerados com asma os adolescentes que apresentaram chiado no peito nos últimos 12 meses. Um modelo logístico múltiplo foi ajustado para controle do confundimento. O valor p≤0,05 foi eleito para determinar associação estatisticamente significante.

**Resultados:**

Foram estudados 106.983 adolescentes. A prevalência de asma foi de 23,2%. O modelo final foi composto de 11 variáveis, que se associaram à asma de forma independente e estatisticamente significante (p<0,001): sexo feminino (OR=1,17), idade inferior a 14 anos (OR=1,12), não morar com os pais (OR=1,06), o maior número de dias de consumo de alimentos ultraprocessados (OR=1,16), almoçar ou jantar sem a presença dos pais ou responsáveis (OR=1,13), realizar as refeições em frente à TV ou estudando (OR=1,18), não tomar café da manhã com frequência (OR=1,22), ter fumado cigarro (OR=1,36), ter experimentado bebida alcoólica (OR=1,37), ter usado droga ilícita (OR=1,29) e ter procurado serviço de saúde no último ano (OR=1,67).

**Conclusões:**

Os resultados do presente estudo reforçam a característica multifatorial de determinação da asma. Estratégias de prevenção e controle devem focar grupos de adolescentes que vivem em condições inadequadas do ponto de vista familiar, alimentar e comportamental (uso de drogas).

## INTRODUCTION

Asthma is a chronic multifactorial disease characterized by variable limitation of airflow and inflammation of the lower airways, leading to respiratory symptoms such as coughing, wheezing, and shortness of breath. Its is associated with genetic and environmental factors, which play a key role in the intensity of clinical manifestation.^1^ Asthma has been considered a public health problem due to the increase in the number of cases worldwide. In Brazil, prevalence rates are high, especially in childhood and adolescence, life phases in which it is considered to be the most common chronic noncommunicable disease.^2^ ^,^ ^3^ ^,^ ^4^

The last edition of the International Study of Asthma and Allergies in Childhood (ISAAC, phase III) identified an average prevalence of 19% of active asthma in Brazilian children and adolescents, while the second version of the National School Health Survey (PeNSE), conducted in 2012, estimated a prevalence of 23.2% among Brazilian schoolchildren in the 9^th^ year of elementary school, based on the same questions from ISAAC.^5^ ^,^ ^6^ ^,^ ^7^ The PeNSE-2012, a Brazilian survey representing adolescents, was the first national initiative that gathered epidemiological characteristics of various levels of the disease process determination,^6^ but no research has been conducted on asthma-related factors, which could potentially guide health strategies for its control and prevention in this population group.

Several factors related to asthma in adolescents have been identified in recent years. Among them, lifestyle changes in recent decades, which have led to complex environmental, behavioral and dietary changes, have been pointed as important determining factors for the onset of asthma.^7^ ^,^ ^8^ ^,^ ^9^ Biological, cognitive, emotional and social changes, which are characteristics of adolescence, can lead to greater exposure to various health risk factors, such as smoking, alcohol consumption, inadequate diet and sedentary lifestyle. In addition, this phase of life is favorable for the adoption of new practices, acquisition of knowledge, changes in behavior and autonomy gain.^6^

This multifactorial aspect of asthma paves the way to investigations of factors associated in the various hierarchical levels of its determination, including socioeconomic, demographic, feeding, environmental, behavioral and health characteristics. Thus, the objective of the present study was to identify factors associated with asthma among Brazilian schoolchildren of the 9^th^ year of elementary school who took part in PeNSE-2012.

## METHOD

This study used secondary data from PeNSE-2012, which was a cross-sectional health survey conducted by the Brazilian Ministry of Health in partnership with the Brazilian Institute of Geography and Statistics (IBGE).^6^

The target population were schoolchildren enrolled in the 9^th^ year of elementary school, in day shift, at public and private schools located in urban or rural areas of Brazil. The sample was assembled to estimate population parameters (proportions or prevalence) and represent 32 geographic strata: 26 State capitals, the Federal District and the five macroregions of Brazil, composed of the other municipalities. The sample was then composed of 109,104 subjects. Other information on geographic stratification, process of school and 9^th^ grade groups selection, and allocation of schoolchildren are described in the PeNSE-2012 report.^6^

Data from 108,350 schoolchildren who answered the questioning chosen to define the outcome variable (“wheezing in the last 12 months”) were analyzed, as, according to the ISAAC protocol, this is the most sensitive and specific factor for its diagnosis,^10^ leading to initial sample loss of 0.7% in the univariate analysis. In addition, 1,367 schoolchildren without information on variables composing the final multiple regression model were excluded. Therefore, 106,983 adolescents were studied, totaling a sample loss of 1.9%.

Data was collected from April to September 2012 through self-completion of a structured questionnaire in a smartphone, with 127 questions organized in 15 thematic modules which included information on demographic, socioeconomic, clinical and environmental characteristics of participants.^6^

A variable was constructed to represent the intake of traditional, fresh food (beans, raw vegetables, raw salads, salads and cooked vegetables, fruits and milk), and another to represent the intake of ultra-processed food (fried pastries, sausages, crackers, cookies, salty snacks, snacks in general and soda), based on the average number of days of intake in the last week defined for each category. The score assigned to each variable was between 0 and 7.

For the definition of the variables related to history of drug use (cigarettes, alcoholic beverages and illicit drugs), questions that could draw the use of such substances at least once in life were defined

Data were evaluated according to their distribution characteristics, and cut-off points were defined as per what had been previously used in the literature and by causal plausibility. Descriptive statistics were used to study associations. The statistical test used to measure associations was the chi-square, as variables studied were parametric. Subsequently, a logistic regression model was adjusted to independently identify factors associated with asthma. For the selection of independent variables eligible to compose the final multiple model, p≤0.20 was considered. The variable input technique was Stepwise Forward, and p≤0.05 was defined as indicative of statistically significant association. The statistical analysis was carried in Stata 14.0. All analyses were based on the expansion technique and sample weight according to PeNSE-2012 selection process and population representativeness.

Students who voluntarily agreed and signed the free and informed consent form took part in the research. PeNSE-2012 was approved by the National Research Ethics Committee (CONEP) (Registration No. 16,805). Although the present investigation used secondary data from PeNSE-2012, the analyses began only after the approval by the Research Ethics Committee of *Universidade Federal de São Paulo* (UNIFESP), opinion 0262/2017.

## RESULTS

Table 1 lists the descriptive characteristics of students: 44.3% of them were from the Southeast region, 52.2% were females, 82.8% were enrolled in a public school, 68.5% were under 14 years of age and 62.2% lived with their parents.

**Table 1:** Prevalence and respective 95% confidence intervals of clinical and epidemiological characteristics of 9^th^ grade students in the study, National School Health Survey of 2012.

Characteristics		n	%	95%CI
Socioeconomic/demographic				
Macro-region	North	108,350	8.0	7.5-8.4
	Northeast		25.3	23.7-27.1
	Mid-West		7.9	6.7-9.2
	Southwest		44.3	41.6-47.0
	South		14.6	13.4-15.8
School	Public	108,350	82.8	77.8-86.9
	Private		17.2	13.1-22.2
Child’s sex	Female	108,350	52.2	50.2-54.3
	Male		47.8	45.7-49.8
Child’s ethnicity	Others	108,296	63.2	57.8-68.3
	White-Caucasian		36.8	31.7-42.2
Child’s age	≤14 years	108,350	68.5	59.4-76.4
	>14 years		31.5	23.6-40.6
Living with parents	No	108,191	37.8	35.9-39.8
	Yes		62.2	60.2-64.2
Mother’s education	No study	90,035	10.0	8.4-12.0
	Study		90.0	83.0-91.6
Father’s education	No study	83,539	15.2	13.1-17.4
	Study		84.8	82.6-86.9
Eating habits				
Intake of fresh food in the past 7 days	<5 days	107,792	82.1	80.9-83.3
	≥5 days		17.9	16.7-19.1
Intake of ultra-processed food in the past 7 days	>2	107,792	64.4	63.0-65.8
	≤2		35.6	34.2-37.0
Lunch time or dinner time in the presence of parents or guardians in the past week	≤4 days	108,281	28.5	26.5-30.5
	>4 days		71.5	69.5-73.5
Meals in front of the TV or while studying	Yes	108,221	81.2	78.7-83.4
	No		18.8	16.6-21.3
Breakfast in the past week	≤4 days	108,251	38.1	35.8-40.5
	>4 days		61.9	59.5-64.2
Drug use history				
Cigarette	Yes	108,329	19.6	17.1-22.3
	No		80.4	77.7-82.9
Alcohol	Yes	108,350	66.6	64.0-69.2
	No		33.4	30.8-36.0
Illicit drugs	Yes	108,327	7.0	5.3-9.4
	No		93.0	90.6-97.7
Respiratory problems				
History of asthma*	Yes	108,115	12.4	11.4-13.5
	No		87.6	86.5-88.6
Health service history				
Has sought health care service in the last 12 months	Yes	108,227	48.2	46.1-50.2
	No		51.8	49.8-53.9

95%CI: 95% confidence interval; *diagnosis by a physician at some point in their life.

The intake of traditional, fresh food less than five days in the last week before filling in the questionnaire was 82.1%, while the intake of ultra-processed food more than twice a week was 64.4%. The frequency of lunch or dinner in the presence of their parents or guardians four or less times in the week was 28.5%, the habit of having meals in front of the TV or studying was 81.2%, and the frequency of breakfast four times or less in a week was 38.1%. In addition, 19.6% of them had tried cigarettes, 66.6% had tried alcoholic beverages, and 7.0% had used illicit drugs. More specifically, 12.4% of the sample was diagnosed with asthma at some time in their lives, and 48.2% had sought healthcare services in the last 12 months (Table 1).

**Graph 1:**
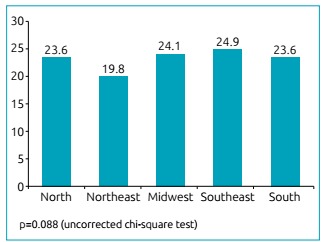
Comparison of prevalence (%) of asthma in 9^th^ grade students between the five Brazilian macro regions, National School Health Survey of 2012.

According to the question related to active asthma, the prevalence of wheezing in the last year in Brazil was 23.2% (95% confidence interval - 95%CI 21.2-25.4). Graph 1 shows the prevalence values per the five Brazilian macro regions. The Southeast Region had the highest prevalence (24.9%), while the Northeast had the lowest (19.8%); however, there was no statistically significant difference between them (p=0.088).

Table 2 presents the bivariate and multiple analyses of factors associated with active asthma. The final logistic regression model, controlled by Brazil’s macroregion and the medical diagnosis of asthma at some point in life, was composed of 11 variables, which remained independently and statistically significant (p<0.001).

**Table 2:** Prevalence, *Odds Ratio*/adjusted *Odds Ratio* and respective 95% confidence intervals of factors associated with active asthma in schoolchildren of the 9th grade, National School Health Survey of 2012.

Characteristics		%	OR (95%CI)	p-value	Adjusted OR (95%CI)	p-value
Socioeconomic/demographic						
School	Private	24.8	1.12 (1.07-1.17)	<0.001		
	Public	22.7				
Sex	Female	24.6	1.19 (1.13-1.25)	<0.001	1.17 (1.12-1.22)	<0.001
	Male	21.5				
Ethnicity	White-Caucasian	23.2	1.01 (0.97-1.03)	0.60		
	Others	23.1				
Age	≤14 years	23.3	1.04 (1.00-107)	0.046	1.12 (1.08-1.15)	<0.001
	>14 years	22.7				
Living with both parents	Yes	21.9	1.18 (1.15-1.21)	<0.001	1.06 (1.03-1.08)	<0.001
	No	24.9				
Mother is educated	Yes	23.7	1.10 (1.03-1.16)	<0.001		
	No	22.0				
Father is educated	Yes	23.7	1.06 (1.00-1.12)	<0.001		
	No	22.7				
Eating habits						
Intake of fresh food in the past 7 days	<5 days	23.2	1.04 (0.90-1.09)	0.144		
	≥5 days	22.6				
Intake of ultra-processed food in the past 7 days	>2 days	24.9	1.33 (1.28-1.37)	<0.001	1.16 (1.11-1.19)	<0.001
	≤2 days	20.0				
Lunch time or dinner time in the presence of parents or guardians in the past week	≤4 days	25.9	1.24 (1.20-1.28)	<0.001	1.13 (1.08-1.16)	<0.001
	>4 days	21.9				
Meals in front of the TV or while studying	Yes	24.0	1.30 (1.24-1.35)	<0.001	1.18 (1.13-1.22)	<0.001
	No	19.6				
Breakfast in the past week	≤4 days	26.5	1.36 (1.32-1.40)	<0.001	1.22 (1.17-1.26)	<0.001
	>4 days	20.9				
Drug use history						
Cigarette	Yes	31.2	1.71 (1.64-1.77)	<0.001	1.36 (1.30-1.41)	<0.001
	No	21.0				
Alcohol	Yes	26.1	1.67 (1.59-1.74)	<0.001	1.37 (1.31-1.44)	<0.001
	No	17.0				
Illicit drugs	Yes	34.4	1.85 (1.76-1.92)	<0.001	1.29 (1.22-1.36)	<0.001
	No	22.2				
Health service history						
Has sought health care service in the last 12 months	Yes	27.9	1.71 (1.65-1.76)	<0.001	1.67 (1.62-1.72)	<0.001
	No	18.5				

OR: *Odds Ratio*; 95% CI: 95% confidence interval. Final logistic regression model controlled by macro-region and diagnosis of asthma at some point in life.

## DISCUSSION

As per results described in PeNSE-2012, our study estimated that approximately one in four adolescents in the sample had wheezing in the past 12 months (23.2%). Although investigations have shown different rates of asthma in different areas of Brazil, we did not find a statistically significant difference between the five macro-regions of the country.^5^ ^,^ ^11^

This national estimate was higher than the rates found in ISAAC phases I (1996) (22.7%) and III (2003) (19%), as well as in the Cardiovascular Risk Study in Adolescents (ERICA, 2016) (13.1%).^5^ ^,^ ^11^ ^,^ ^12^ This increase is probably explained by the reduction in exposure to early-life infections due to improved sanitary conditions in recent decades.^13^ On the other hand, the decrease reported in ERICA can be associated with the wider age range of the group evaluated (12 to 17 years), which included older adolescents. In addition, the original question in ISAAC on active asthma used in PeNSE-2012, whose sensitivity was 88% and specificity was 90%, was reformulated in ERICA, with the replacement of the term wheezing with wheezing episodes. This change may have reduced the sensitivity to identify asthmatic adolescents.^11^

Also, the methods used allowed to identify 11 factors independently associated to this event: being a female, age under 14 years, not living with parents, larger number of days of consumption of ultraprocessed foods, having lunch or dinner without the presence of parents or guardians, eating meals in front of the TV or while studying, frequently not having breakfast, smoking cigarettes, drinking alcohol, use of illicit drugs, and search for health care in the last year.

The instrument for data collection developed and applied by ISAAC, which allowed epidemiological investigations and comparisons of prevalence rates, also allowed to identify asthma-related factors among children and adolescents in several countries. In this sense, changes in lifestyle, environmental pollution, dietary changes, smoking, exposure to allergens, and better hygiene conditions were factors associated with asthma in the last decades.^7^ ^,^ ^8^ ^,^ ^9^ ^,^ ^10^

Predominantly, studies have indicated that the prevalence of asthma in adolescence is higher in females, which is in agreement with our findings, although there is not a well-established cause for this association at this time.^4^ ^,^ ^14^ On the other hand, Lima et al.^15^ did not report a statistically significant difference in prevalence of asthma between sexes of adolescents from São Luís do Maranhão. The higher prevalence of asthma in female adolescent can be explained by the influence of hormonal factors, environmental exposures typical of each sex, and possible differences in the way of filling the questionnaire, since boys tend to underestimate symptoms and girls usually overestimate them.^16^ ^,^ ^17^

Adolescents aged less than or equal to 14 years were at increased risk of asthma. In the pediatric age group, asthma is more frequent in the first years of life, following a natural history of symptom remission over the years and in the second decade of life. As adolescents in younger age groups are in the initial period of disease control, this may justify the association found.^18^

The association between eating habits and asthma has been progressively studied in order to understand the possible characteristics of food consumption that favor its development.^19^ A recent meta-analysis suggested a possible protective effect of high consumption of fruits and vegetables against asthma and respiratory diseases, although there is a gap in the literature when it comes to the biological response mechanism of disease control and development.^20^ Low consumption of traditional fresh food, such as fruits and vegetables, and increased consumption of ultraprocessed food reflect the consequences of globalization and the transition to the western diet in the last decades, which has established new paradigms and changes in food choices.^8^ ^,^ ^9^ Our study shows a risky association between the intake of ultraprocessed foods and asthma. In fact, reduced consumption of antioxidants (vitamins A, E and C), minerals (zinc, selenium, copper) and bioactive compounds, which all have a protective action on the respiratory system and are less frequent in ultraprocessed foods, is a consequence of this change in food standard.^19^ ^,^ ^20^

The association between the habit of eating in front of the TV and asthma regardless of the nutritional quality indicators used here was confirmed. Despite this, this habit is related to the increase in the consumption of food with higher energy density and low nutritional value, since the distraction caused by screens interferes with the physiological signs of hunger and satiety, leading to inadequate feeding choices.^21^ In this sense, eating in front of the TV would be associated with a greater risk of asthma due to other phenomena involved in this behavior, but not characterizing direct effect on the greater risk of asthma.

The absence of parents or guardians at the time of main meals also jeopardizes the quality of feeding, as there is no example of caregivers with healthy eating habits, which allows adolescents to make inadequate food quality choices.^22^ In the same way, not having breakfast is related to the lower average consumption of fibers and micronutrients, reflecting a decrease in the consumption of food that is source of antioxidants, important for the development and control of asthma.^23^

Although the association between smoking and asthma is well studied, there are few studies evaluating active smoking in adolescence versus respiratory diseases.^24^ Adolescents studied by PeNSE-2012 who smoked cigarettes had an increased risk of asthma, which corroborates the results of other studies with children and adolescents that have shown that smoking cigarette is more frequent among asthmatics and that smoking is one of the factors increasing the risk of persistent diseases in adulthood.^24^ ^,^ ^25^ Smoking may not be the cause of the development of asthma in adolescence, but it may increase its persistence. In general, asthma occurs before the exposure to active smoking, a habit that usually begins at puberty.^2^ ^,^ ^18^ ^,^ ^24^ Likewise, the use of illicit drugs was associated with asthma. Despite the fact that the acute inhalation of cannabis (marijuana), the illicit drug most commonly consumed by adolescents worldwide, contributes to improvement in airflow due to the bronchodilation it produces, there is a recognized association between its chronic use and central airway inflammation and compromising of pulmonary function.^26^ ^,^ ^27^ Research shows a possible synergy and predisposition to a combination of effects between tobacco and cannabis on lung function. Despite this, the risk of asthma among adolescents who smoked cigarettes and used illicit drugs were independently identified.^27^

Despite the common association between smoking and alcoholic beverages, alcohol consumption in our study was associated with asthma regardless of smoking. Although several effects of level of consumption and type of alcoholic beverage on pulmonary function have already been identified, PeNSE-2012 does not provide this information, which impedes a safe interpretation of findings related;^28^ however, alcohol consumption suppresses Th1-dependent immune response to allergens and distorts Th2 response, which leads to increased production and release of cytokines and immunoglobulin E (IgE). Therefore, individuals who make high alcohol consumption are at higher risk for allergic respiratory diseases.^29^

For being chronic, asthma leads to a greater need for follow-up in both primary and early care. Thus, adolescents assessed by PeNSE-2012 who presented with wheezing in the past 12 months sought health services more frequently.^30^

It should be noted that using secondary data limited the analyses, since only information available from PeNSE-2012 was used. Family history, nutritional status and presence of comorbidities, which are factors commonly associated with asthma, were not evaluated in this study.^15^ This may have partially compromised the results obtained, since some factors, such as eating in front of the TV, may represent other determinant characteristics of asthma that have not been tested, such as being overweight. This possibility leads to the risk of false associations, characterized by being significant, but resulting from chance.

Also in this sense, data collection through a self-administered questionnaire probably led to a greater risk of errors in estimates of information collected. The imprecise measure in some questions may have limited the findings. Moreover, although they are statistically significant, the associations do not allow establishing relations of causality with safety, since this is a cross-sectional study and should be interpreted with caution, because the magnitude of effect achieved was low.

On the other hand, PeNSE-2012 was a survey aimed at the population of adolescents enrolled in the 9^th^ year of elementary education in Brazil that used a judicious process of selection of participating schools and, consequently, allowed the recruitment of a sample representative of the national territory. Another important aspect is the question of the ISAAC protocol for asthma, which enabled the investigation of the disease in Brazil for international comparison of data. In addition, the multiple regression model allowed to control confounding factors when estimating associations, thus identifying the independent effect of all 11 factors related to this respiratory disease.

In this context, our results point to factors associated with asthma in adolescence, corroborating with its multifactorial aspect that involves characteristics of different hierarchical levels of schoolchildren. Therefore, despite the limitations, these factors must be taken into account when developing asthma prevention and control strategies.

In summary, these findings suggest that Brazilian adolescents living in inadequate familial, feeding and behavioral conditions (drug use) are more likely to have active asthma.
